# Recent Developments in Flexible Thermoelectric Devices

**DOI:** 10.1002/smsc.202100005

**Published:** 2021-05-13

**Authors:** Shiqi Yang, Pengfei Qiu, Lidong Chen, Xun Shi

**Affiliations:** ^1^ State Key Laboratory of High Performance Ceramics and Superfine Microstructure Shanghai Institute of Ceramics Chinese Academy of Sciences Shanghai 200050 China; ^2^ Center of Materials Science and Optoelectronics Engineering University of Chinese Academy of Sciences Beijing 100049 China; ^3^ School of Chemistry and Materials Science Hangzhou Institute for Advanced Study University of Chinese Academy of Sciences Hangzhou 310024 China

**Keywords:** flexible thermoelectric, power density, self-powered technology, wearables

## Abstract

Flexible thermoelectrics, including flexible thermoelectric materials and devices, can directly convert the heat from human body into useful electricity, providing a promising solution for uninterrupted power to wearables. In the past decade, flexible thermoelectrics has achieved notable progress. Various kinds of flexible thermoelectric materials have been developed and some of them have been fabricated into flexible thermoelectric devices, showing the ability to generate nW‐level or even μW‐level electricity. Herein, the basic design principles and typical configurations of flexible thermoelectric devices, as well as the requirements on thermoelectric materials to achieve high performance flexible thermoelectric devices, are first introduced. Then, the recent progress achieved in flexible thermoelectric devices based on organics materials, traditional inorganic materials, other organic/inorganic composites/hybrids, and plastic deformable inorganic semiconductors, respectively, are summarized. Finally, an outlook on the future development of flexible thermoelectrics is briefly given. This study sheds light on the further development of flexible thermoelectrics.

## Graphical abstract

This review systematically summarizes 1) the basic design principles and typical configurations of flexible thermoelectric devices, 2) the requirements on thermoelectric materials to achieve flexible thermoelectrics, and 3) the recent progress in flexible thermoelectric devices based on different kinds of thermoelectric materials. Finally, this review briefly gives an outlook on the future development of flexible thermoelectrics.

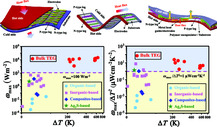

## 1 Introduction

Wearables is a category of electronic devices that can be worn as accessories, embedded in clothing, implanted in the user's body, or even tattooed on the skin. The past decade has witnessed rapid development of wearables in medicine, healthcare, military, and other fields. As predicted by global technology research firm CCS Insight, the market of wearables will reach almost $30 billion and the sales will reach 260 million units in 2023.^[^ ^1^ ^]^

The rapid development of wearables is originated from the great successes achieved in materials, electronics, and other related techniques. Inversely, it also proposes more requirements to them. One typical example is the battery for the wearables. The appearance of portable chemical battery, especially the ultrathin large capacity lithium battery, makes the service of wearables possible. Inversely, the rapid development of wearables requires the batteries have better flexibility, smaller volume, larger capacity, and longer lifetime.^[^ ^2^ ^]^ Among them, the longer lifetime of battery is one of the most important issues because the frequently charging process greatly limits the long‐term service of wearables, which is particularly critical for the applications in medicine and military fields.

Beyond the traditional chemistry battery, self‐powered technology is attracting increasing attention in both academic and industry fields because it can provide uninterruptable power supply to wearables.^[^ ^3^ ^]^ The most promising self‐powered technology is thermoelectric technique, which can directly generate power by using the temperature difference between human body and environment based on the Seebeck effect.^[^ ^4^ ^]^ It has the advantages of silent, reliable, and without moving parts. As early as 1960s, thermoelectric technique has been already used to power the deep space satellite to perform long‐term exploration in deep space. Since the last decade of 20th century, due to the energy crisis and aggravated greenhouse gas emissions, the applications of thermoelectric technique were extended to the fields of waste heat harvesting.^[^ ^5^ ^]^ In these applications, the heat sources usually have large area and thus the rigid thermoelectric materials and planar devices can satisfy the requirements. However, in wearables, the thermoelectric devices should be flexible to well fit the curved surface of human skin and survive under frequently bending process. This makes the development of flexible thermoelectric devices a very challenging task. Currently, flexible thermoelectrics, including flexible thermoelectric materials and devices, has already become a hot topic in thermoelectric community. The number of published academic studies included in Web of Science Database (WOS) relevant to the keywords “flexible thermoelectric material” and “flexible thermoelectric device” is continuously increasing, from 18 in 2010 to 205 in 2019.

Flexible organic thermoelectric materials are natural candidates for flexible thermoelectric devices due to their intrinsic flexibility.^[^ ^6^ ^]^ Likewise, via the help of flexible substrates^[^ ^7^ ^]^ or deformable interconnectors,^[^ ^8^ ^]^ brittle inorganic thermoelectric materials can be also fabricated into flexible thermoelectric devices. The flexible organic/inorganic composites/hybrids^[^ 9, 10 ^]^ and recently discovered plastic deformable inorganic semiconductors^[^ 11, 12 ^]^ provide new options to fabricate the flexible thermoelectric devices. Currently, there are already several excellent reviews which comprehensively summarized the progress of flexible thermoelectric materials.^[^ 13, 14, 15, 16, 17 ^]^ For example, Du et al.^[^ ^13^ ^]^ and Wang et al.^[^ ^14^ ^]^ reviewed the recent development of thermoelectric materials that can be used to fabricate flexible thermoelectric devices. Blackburn et al. reviewed the carbon nanotube (CNT)‐based thermoelectric materials and devices.^[^ ^16^ ^]^ Du et al. reviewed the role of additive manufacturing on fabricating flexible thermoelectric materials and devices.^[^ ^17^ ^]^ In this review, we summarize the recent progress made in the development of flexible thermoelectrics from the point view of device. We first introduce the basic design principles and typical configurations of flexible thermoelectric devices, as well as the requirements to thermoelectric materials by flexible thermoelectrics. Then, we summarize the recent progress achieved in flexible thermoelectric devices based on organics materials, traditional inorganic materials, organic/inorganic composites/hybrids, and plastic deformable inorganic semiconductors, respectively. Particularly, the pros and cons of different flexible thermoelectric materials are compared in the device level. Finally, we briefly give an outlook on the future development of flexible thermoelectrics.

## 2 Basic Device Design Principles

Being the same with traditional rigid thermoelectric devices, the maximum energy conversion efficiency (*η*_max_) for flexible thermoelectric devices in the ideal case can be defined as

(1) $$\eta_{\text{max}}=\frac{T_{H}-T_{C}}{T_{H}}\cdot \frac{\sqrt{1+\bar{ZT}}-1}{\sqrt{1+\bar{ZT}}+\frac{T_{C}}{T_{H}}}$$

where *T*_H_ and *T*_C_ refer to the hot‐side temperature and cold‐side temperature, respectively. $\bar{ZT}$ represents the average thermoelectric figure of merit (*ZT*) between *T*_H_ and *T*_C_. *ZT* is defined as

(2) $$ZT=\frac{\alpha^{2}}{R\cdot K}\cdot T$$

where *α*, *R*, and *K* are Seebeck coefficient, electrical resistance, and thermal conductance of one thermoelectric p/n couple, respectively. *T* is the average value of *T*_H_ and *T*_C_. From the point view of material, the thermoelectric material with large Seebeck coefficient *S*, low electrical resistivity *ρ* (or high electrical conductivity *σ*), and low thermal conductivity *κ* are prone to realize high $\bar{ZT}$. Thus, to maximize the *η*_max_, the thermoelectric materials used in the flexible thermoelectric devices with high *zT* (=*S* ^2^ *T/ρκ*) are necessary. In addition, the open‐circuit voltage (*V*_OC_) and power output (*P*_out_) for the flexible thermoelectric devices are listed as follows

(3) $$V_{\text{OC}}=N\alpha \cdot (T_{H}-T_{C})$$

(4)

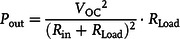

where *R*_in_ is the internal resistance of device, *R*_Load_ is the resistance of applied load in the circuit, and *N* is the number of p/n couples. When *R*_in_ = *R*_Load_, the *P*_out_ reaches the maximum value
(5)

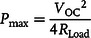




As the flexible thermoelectric devices are usually used to generate electricity by using the heat from human body, which are unlimited and free of cost, the maximum power density (*ω*_max_) is more important for the real application than the *η*_max_. In the ideal case, assuming that *ρ = ρ*_n_ *= ρ*_p_, *ω*_max_ can be given by

(6) $$\omega_{\text{max}}=\frac{P_{\text{max}}}{A_{D}}=\frac{{\left(T_{H}-T_{C}\right)}^{2}}{4L}\cdot \frac{\alpha^{2}\cdot f}{\rho }$$

where *A*_D_ is the cross‐sectional area of the device and *f* is the fill factor (the ratio of the total area of legs to *A*_D_). Under fixed *T*_H_, *T*_C_, *f*, and thermoelectric legs’ length *L*, *ω*_max_ only replies on the thermoelectric materials’ *S* and *ρ*. Thus, to maximize the device's performance, the thermoelectric materials used in the flexible thermoelectric devices should possess high *PF* (=*S* ^*2*^ */ρ*). To this end, the inorganic thermoelectric materials are superior to organic thermoelectric materials because the former usually possesses high carrier mobilities that are several orders of magnitude higher than the latter, which facilities the realization of high *PF*.

Although the thermal conductivity *κ* is not directly included in Equation (6), it should be noted that neglecting the effect of thermal conduction in *ω*_max_ is one‐sidedness. Considering the low quality of heat provided by human body and the small *L*, maintaining the required temperature difference Δ*T* = (*T*_H_ – *T*_C_) across the flexible thermoelectric devices is a very important but critical task. According to the Fourier's law of heat conduction, the power density *E* required to build a Δ*T* on a material with thickness *L* can be expressed as

(7) E=ΔT⋅κ/L

Clearly, when the geometry and heat source are fixed, the magnitude of Δ*T* is determined by the material's thermal conductivity *κ*. Thus, beyond the high *PF*, low *κ* is necessary for the thermoelectric materials used in the flexible thermoelectric devices to build the required Δ*T*. To this end, the organic thermoelectric materials are superior to the inorganic thermoelectric materials because they usually possess very low *κ*.

Beyond the materials’ inherent electrical transport properties, the geometry of flexible thermoelectric devices will also influence the performance. The *V*_OC_ is a geometry‐independent parameter, but the *R*_in_ is directly determined by the geometry. The thermoelectric legs possessing large *A* and/or small *L* will result in low *R*_in_ and consequently large *ω*_max_. The relation between *η*_max_ and geometry is more complex. Too large *A* and/or small *L* will simultaneously result in high thermal conductance *K* and deteriorate the *η*_max_. Thus, proper geometry should be chosen to make a trade‐off between *R*_in_ and *K* according to the working conditions.

It should be noted that Equation (1) and (6) are derived in the ideal case just considering the thermoelectric materials’ intrinsic electrical and thermal transport properties. In the real case, many other factors will influence the actual *η* and *ω*. Among them, the contact electrical/thermal resistances at the interfaces are the most critical factors because they will induce additional energy loss at the interfaces. To maximize the *η* and *ω*, the contact electrical/thermal resistances should be as small as possible.

## 3 Requirements to Flexible Thermoelectric Materials

High‐performance flexible thermoelectric devices require thermoelectric materials possessing good flexibility and high *zT*s. Organic thermoelectric materials (e.g., poly(3,4‐ethylenedioxythiophene):poly(styrene sulfonate) (PEDOT:PSS),^[^ ^18^ ^]^ poly(3‐hexylthiophene) (P3HT),^[^ ^19^ ^]^ polyethyleneimine (PEI),^[^ ^20^ ^]^ and polyaniline (PANI)^[^ ^21^ ^]^) have intrinsically good flexibility, low thermal conductivity, lightweight, and environmental friendliness, but high electrical resistance *ρ* and low power factor *PF*s. Due to the low carrier mobilities, their *PF*s are orders of magnitude lower than those of inorganic materials, which severely restricts the power output of flexible thermoelectric devices. In contrast, the inorganic thermoelectric materials (e.g., Bi_2_Te_3_,^[^ 22, 23, 24 ^]^ PbTe,^[^ 25, 26, 27, 28 ^]^ SiGe,^[^ 29, 30 ^]^ filled skutterudites,^[^ 31, 32 ^]^ half‐Heusler alloys,^[^ 33, 34, 35, 36 ^]^ and Cu_2_Se^[^ 37, 38, 39, 40 ^]^) have high carrier mobilities and thus high *PF*, but they are difficult to satisfy the good bendability, foldability, and stretchability required by a flexible thermoelectric device due to their intrinsic brittleness. Only when they are made into low‐dimension films or their microstructure is specially designed, they can gain some flexibility. The organic/inorganic composites/hybrids, particularly CNT‐based composites/hybrids, have the possibility to simultaneously realize good flexibility and reasonable *zT*s and thus they are one kind of potential candidates to fabricate flexible thermoelectric devices. In addition, the recently developed plastic deformable inorganic semiconductors (e.g., Ag_2_S,^[^ ^11^ ^]^ ZnS,^[^ ^41^ ^]^ and InSe^[^ ^42^ ^]^) provide a new and powerful strategy to flexible thermoelectrics. According to the species of thermoelectric materials used wherein, the flexible thermoelectric devices reported so far can be roughly divided into four classes, termed 1) organic materials‐based, 2) inorganic materials‐based, 3) organic/inorganic composites‐based, and 4) plastic deformable inorganic semiconductors‐based. The organic materials/hybrids, organic/inorganic composites/hybrids, and plastic deformable inorganic semiconductors with good flexibility can be directly fabricated into flexible thermoelectric devices. The brittle inorganic materials need to be first deposited in the form of thin film or connected by deformable interconnectors, and then assembled into device to gain some flexibility in device level.

In thermoelectric devices, the thermoelectric materials need to be connected electrically in series and thermally in parallel by metal electrodes. Particularly, the flexible thermoelectric devices need to experience frequently bending or stretching during service. Thus, beyond the good flexibility and high *zT*s, the thermoelectric materials used in flexible thermoelectric devices should be able to form low energy loss and robust interfaces with the electrodes to maximize the power output and increase the reliability during service. Currently, connecting the thermoelectric materials and electrodes by Ag paste is a common method to assemble the flexible thermoelectric devices considering the high electrical/thermal conductivity of Ag and the easement to realize mass production. However, the contact electrical/thermal resistances and bonding strength between Ag paste and thermoelectric materials are usually not very satisfactory, particularly for the organic materials. New connection methods are still required to be developed in the future.

## 4 Typical Device Configurations

**Figure 1:**
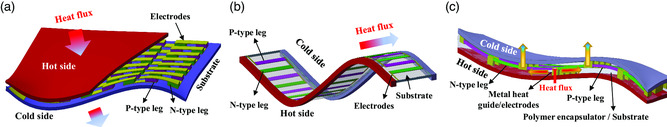
Typical configurations for flexible thermoelectric devices: a) vertical π‐shaped, b) lateral π‐shaped, and c) Y‐shaped.

The reported configurations of flexible thermoelectric devices can be roughly divided into three categories, termed as vertical π‐shaped, lateral π‐shaped, and Y‐shaped. The sketch maps of these three typical configurations are shown in **Figure** **1**. Each flexible thermoelectric device consists of three main components, termed as thermoelectric materials, substrates, and electrodes. Ideally, the thermoelectric materials can be organic materials with intrinsically good flexibility or the inorganic material films that can endure large elastic strain. The substrates can be flexible polymer films (e.g., poly(vinylidene fluoride) [PVDF], poly(dimethyl siloxane) [PDMS], and polyurethane [PU]) or flexible fabrics (e.g., cloth, yarn, silk, polymer fabric, and glass fabric). The electrodes can be flexible metal (e.g., Cu and Ag) foils, pastes, or wires. The n‐type and p‐type thermoelectric materials are connected by electrodes electrically in series and thermally in parallel. The heat flows from the hot end of the device (heat source) to the cold end of the device (heat sink), generating voltage output that empowers the electrons in the external circuit to do work.

In the vertical π‐shaped flexible thermoelectric devices, the n‐type and p‐type thermoelectric legs are vertically adhered on the flexible substrate or embedded inside the flexible substrate. The heat flux direction is perpendicular to the substrate. This configuration is the same with the conventional rigid planar thermoelectric devices. In the lateral π‐shaped flexible thermoelectric devices, the thin thermoelectric legs are laterally attached on the surface of flexible substrate. Being different with the vertical π‐shaped configuration, the heat flux direction is parallel to the substrate. The Y‐shaped flexible thermoelectric devices can be regarded as a synthetic of vertical π‐shaped and lateral π‐shaped flexible thermoelectric devices. The heat from heat source is first harvested in vertical direction through the metal electrodes and then conducted through the thermoelectric legs in lateral direction.

Toward the real applications, the vertical π‐shaped configuration is superior to the lateral π‐shaped configuration because the heat flux direction is usually perpendicular to the substrate instead of parallel to the substrate, such as the case between the human body and environment. In addition, according to Equation (6), theoretically, by adopting short thermoelectric legs to reduce the internal resistance *R*_in_ of the device, the vertical π‐shaped configuration can realize very high *ω*_max_. In contrast, the lateral π‐shaped configuration and Y‐shaped configuration usually have long thermoelectric legs and thus large *R*_in_, which is not benefit for achieving high *ω*_max_. However, the vertical π‐shaped configuration requires both n‐type and p‐type thermoelectric materials. In the case that only one type of thermoelectric materials is available, the flexible thermoelectric device can only adopt the lateral π‐shaped configuration or Y‐shaped configuration, with the unavailable type of thermoelectric materials replaced by the thin metal wires or paste.

## 5 Typical Flexible Thermoelectric Devices

### 5.1 Organic Materials‐Based

The past decades have witnessed great progress achieved in developing high‐performance organic thermoelectric materials. Many conducting polymers, such as PEDOT:PSS, P3HT, PANI, and PEI, demonstrate good thermoelectric performance. Some of them have demonstrated high *zT* exceeding 0.1. However, the conducting polymers are difficult to be doped into n‐type because their electron affinity values are too low to stabilize the n‐type dopants. Thus, up to now, most flexible organic thermoelectric devices have to adopt lateral π‐shaped configuration, in which the thin metal wires or metal pastes are used to directly connect the cold and hot ends of the p‐type thermoelectric legs in series. As there are already several nice reviews about the topic of organic flexible thermoelectric materials,^[^ 43, 44, 45 ^]^ here we only briefly introduce some typical flexible thermoelectric devices based on organic materials.

**Figure 2:**
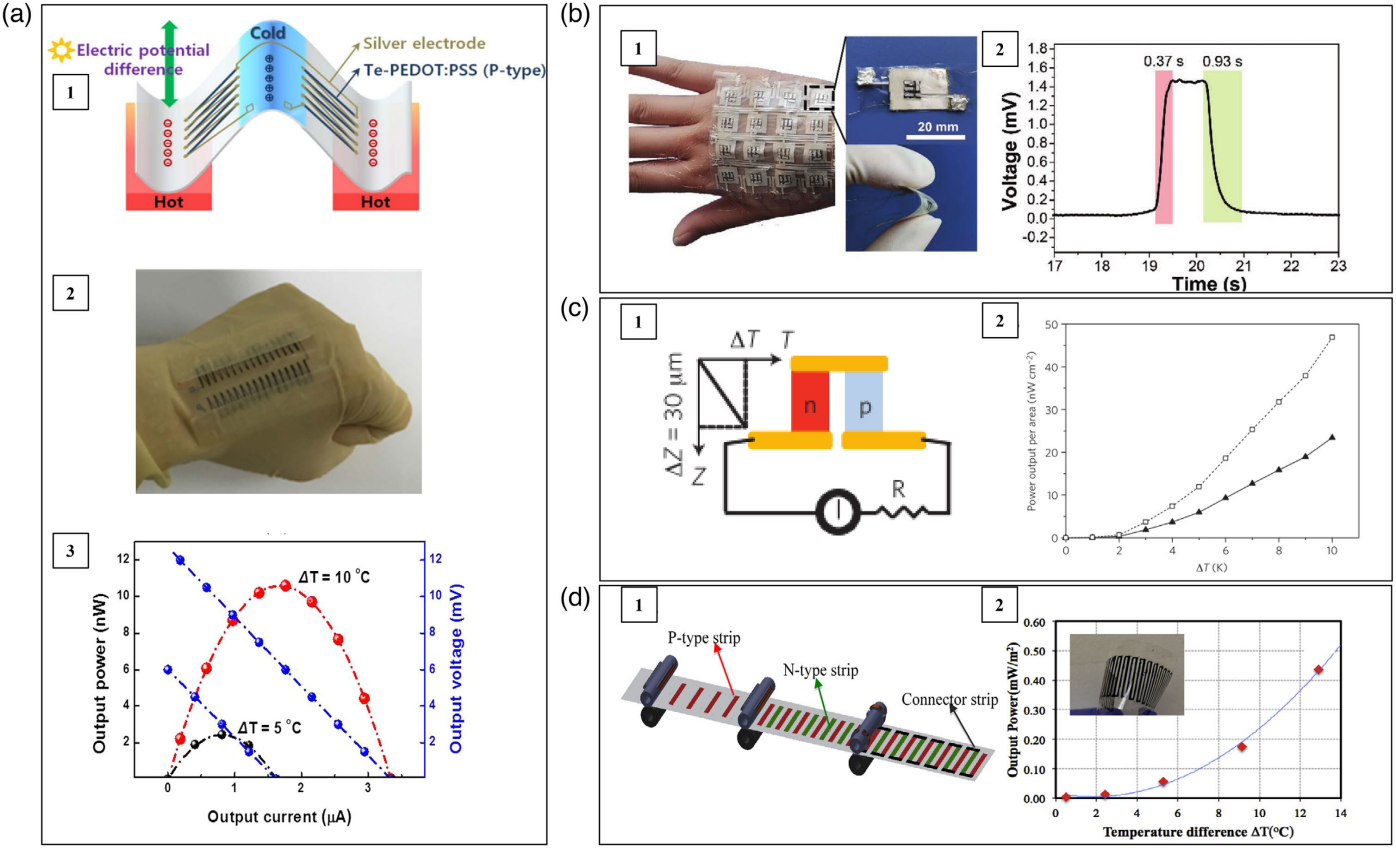
Schematic, optical image, and performance of several flexible thermoelectric devices based on organic thermoelectric materials. a) A 32‐couple device adopting lateral π‐shaped configuration. Reproduced under the terms of the CC‐BY 4.0 license.^[^ ^47^ ^]^ Copyright 2016, The Authors, published by Springer Nature. b) A temperature sensor compromising a flexible thermoelectric device adopting vertical π‐shaped configuration. Reproduced with permission.^[^ ^48^ ^]^ Copyright 2020, Wiley‐VCH. c) A 54‐couple device adopting lateral π‐shaped configuration. Reproduced with permission.^[^ ^50^ ^]^ Copyright 2011, Springer Nature. d) A roll‐to‐roll printed flexible thermoelectric device adopting lateral π‐shaped configuration. Reproduced with permission.^[^ ^51^ ^]^ Copyright 2016, Elsevier.

Søndergaard et al. prepared a flexible thermoelectric device consisting of p‐type PEDOT:PSS thermoelectric material.^[^ ^46^ ^]^ By using the roll‐to‐roll printing approach, bottom Ag interconnections, PEDOT:PSS thermoelectric legs, and top Ag interconnections were successively printed onto the flexible PET substrate. The whole device adopting the lateral π‐shaped configuration included 576 PEDOT:PSS thermoelectric legs. A power output of 43 pW was generated under Δ*T* = 75 K. Bae et al. used p‐type PEDOT:PSS/Te hybrid thermoelectric materials to prepare flexible thermoelectric device, which adopted the lateral π‐shaped configuration (**Figure** **2**a).^[^ ^47^ ^]^ Ag paste was dispensed to connect the 32 p‐type legs electrically in series. A peak power output of 10.5 nW was achieved under Δ*T* = 10 K. Zhu et al. fabricated a device consisting of PANI‐based thermoelectric nanocomposites as p‐type legs, Ag pieces as n‐type legs, and Ag pastes as electrodes.^[^ ^48^ ^]^ As shown in Figure 2b, this device can detect the human body temperature with a high‐temperature sensing sensitivity (109.4 μV K^−1^) and a rapid response time (0.37 s). This study indicated that organic materials‐based flexible thermoelectric devices have a great potential to be used as electronic skin. Kim et al. prepared one kind of flexible thermoelectric device using a selenophene derivative (hexyl‐3,4‐ethyl‐enedioxyselenophene, EDOS‐C6).^[^ ^49^ ^]^ By means of the photothermoelectric effect, the device can convert the light‐driven heat energy into electricity.

The recently developed n‐type small‐molecule organic thermoelectric materials provide the possibility to prepare flexible organic thermoelectric device including n‐type thermoelectric legs. Bubnova et al. built an organic flexible thermoelectric device adopting lateral π‐shaped configuration by alternatively dropping 3,4‐ethylenedioxythiophene (EDOT)/tosylate/pyridine solution (p‐type) and tetrathiafulvalene‐tetracyanoquinodimethane (TTF‐TCNQ)/poly(vinyl chloride) (PVC) blends (n‐type) into epoxy‐based cavities (Figure 2c).^[^ ^50^ ^]^ Au was used as the electrodes to connect n‐ and p‐type thermoelectric legs. A power density around 0.25 mW m^−2^ was achieved under Δ*T* = 10 K.

In flexible thermoelectric device, Ag paste is usually used as electrodes. However, due to the possible oxidation in air and large contact resistance between the organic thermoelectric materials and Ag, the actual internal resistance of the device is usually larger than the theoretical internal resistance. Addressing this issue, Zhang et al. proposed utilizing organic electrodes to replace Ag electrodes.^[^ ^51^ ^]^ They fabricated a device consisting of PEDOT:PSS as p‐type legs, nitrogen‐doped graphene as n‐type legs, and PEDOT:PSS strips as electrodes via roll‐to‐roll printing the corresponding inks on a plastic substrate. PEDOT:PSS thermoelectric legs and PEDOT:PSS electrodes could form a facile dissolution at the interfaces yielding low contact resistance. Consequently, a high power density of 0.24 mW m^−2^ was achieved under Δ*T* = 10 K (Figure 2d).

### 5.2 Traditional Inorganic Materials‐Based

Inorganic thermoelectric materials possess much higher power factors than the organic thermoelectric materials, but their applications in flexible thermoelectric devices are limited by their intrinsic brittleness. Fabricating thin films is one main strategy to realize flexibility in these inorganic thermoelectric materials. In addition, assembling the bulk inorganic thermoelectric materials by deformable interconnectors can also gain some flexibility in device level. Herein, we summarize the progress achieved in flexible thermoelectric devices based on inorganic thermoelectric materials, particularly focusing on those consisting of the Bi_2_Te_3_‐based alloys.

#### 5.2.1 Film‐Based

Inorganic thermoelectric films can be directly deposited on the flexible substates by the chemical or physical methods (e.g., chemical vapor deposition, molecular beam epitaxy, vapor–liquid–solid growth, electrochemical deposition, pulsed laser deposition, and magnetron sputtering), or by printing techniques (e.g., inkjet printing, dispenser printing, and screen printing). Alternatively, they can also be moved to the flexible substates after deposition on the rigid substrate. In special cases, some inorganic thermoelectric materials could also be made into freestanding films. Because the inorganic thermoelectric materials are intrinsically brittle, the thickness of the films is greatly limited. When the films are too thick, the flexibility will be very poor and cracks will be easily generated inside the films or at the interfaces between films and substrates during the bending process. In addition, the application of thin films in vertical π‐shaped flexible thermoelectric devices is greatly limited because the large Δ*T* is difficult to be built across the device. Thus, many inorganic thermoelectric thin films‐based devices have to adopt the lateral π‐shaped or Y‐shaped configurations to maximize the Δ*T*.

Generally, the bulk thermoelectric materials used in the traditional rigid thermoelectric devices possess high relative density and good crystallinity. However, the relative densities of many inorganic thermoelectric films, especially for those fabricated by the chemical methods and printing techniques, are usually not very high. Likewise, being limited by the low melting point of many flexible organic substrates, the as‐printed films are usually annealed at low temperature, yielding poor crystallinity. These issues will deteriorate the electrical conductivity and *zT* in the material level and power output in the device level.

**Figure 3:**
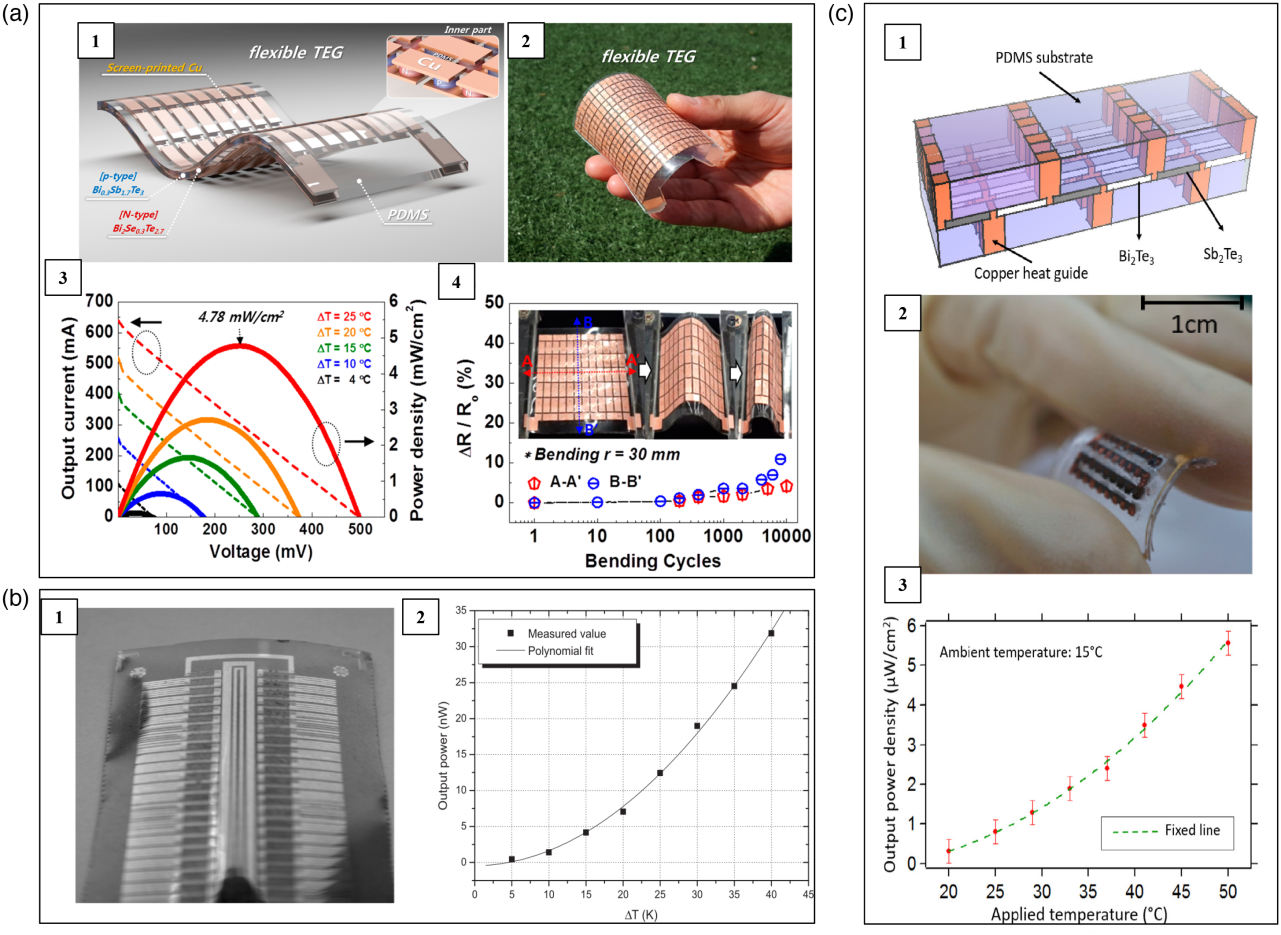
Schematic, optical image, and performance of several flexible thermoelectric devices consisting of Bi_2_Te_3_‐based alloys deposited on polymer substrates. a) A 72‐couple device adopting vertical π‐shaped configuration on a PDMS substrate. Reproduced with permission.^[^ ^52^ ^]^ Copyright 2016, American Chemical Society. b) A 100‐couple device adopting lateral π‐shaped configuration on a Kapton substrate. Reproduced with permission.^[^ ^53^ ^]^ Copyright 2011, Elsevier. c) A 24‐couple device adopting Y‐shaped configuration on a PDMS substrate. Reproduced with permission.^[^ ^56^ ^]^ Copyright 2018, Elsevier.

Kim et al. fabricated a 72‐couple Bi_2_Te_3_‐based film flexible thermoelectric device with vertical π‐shaped configuration by using screen printing and lift‐off process.^[^ ^52^ ^]^ The schematic illustration and the optical image of the device are shown in **Figure** **3**a. The n‐type legs were Bi_2_Se_0.3_Te_2.7_, while the p‐type legs were Bi_0.3_Sb_1.7_Te_3_. Commercial ingots were first crashed into powders by ball milling. Then, the powders were mixed with organic binder and solvent. The paste was screen printed into thermoelectric films on the rigid SiO_2_/α‐Si/quartz substrate with assigned patterns. Ni layer was sputtered on both sides of thermoelectric films as an interfacial layer to reduce the contact resistance between the thermoelectric materials and the Cu electrodes. The product was dipped into PDMS. Finally, a laser multiscanning (LMS) lift‐off technique was used to move the device from the SiO_2_/α‐Si/quartz substrate to form the final freestanding flexible thermoelectric device embedded in PDMS flexible substrate. As shown in Figure 3a, under Δ*T* = 25 K, this device demonstrated a power density of 47.8 W m^−2^. Furthermore, there was no significant change in the device performance even after 8000 bending cycles.

By depositing n‐type Bi_2_Te_3_ and p‐type Sb_2_Te_3_ thin films on Kapton HN polyimide foil via RF magnetron cosputtering technique, Francioso et al. fabricated a thin film flexible thermoelectric device with lateral π‐shaped configuration (Figure 3b).^[^ ^53^ ^]^ The thickness of the films was 500 nm. Gold was sputtered on the thermoelectric materials by mask process to contact the electrodes. Comb‐like gold radiators were also placed on cold junctions to improve heat dissipation and increase Δ*T* between hot and cold junctions. Under Δ*T* = 40 K, this device could generate an open‐circuit voltage of 430 mV and a maximum output power of 32 nW. Wang et al. reported a self‐powered wearable pressure sensing system integrating a Bi_2_Te_3_‐based flexible thermoelectric device.^[^ ^54^ ^]^ The Bi_2_Te_3_ film, Sb_2_Te_3_ film, and Cu electrodes were prepared by magnetron sputtering on a polyimide substrate to form the thermoelectric device adopting lateral π‐shaped configuration. The device can achieve an open‐circuit voltage of 78 mV and a power out of 7.9 μW under Δ*T* = 20 K. By using inkjet printing method, Lu et al. printed the p‐type Sb_1.5_Bi_0.5_Te_3_ nanoparticles, n‐type Bi_2_Te_2.7_Se_0.3_ nanoparticles, and silver electrodes on polyimide to obtain a flexible thermoelectric device with lateral π‐shaped configuration.^[^ ^55^ ^]^ When an electric power was applied onto the device, steady‐state cooling can be realized.

As shown in Figure 3c, Trung et al. fabricated a Bi_2_Te_3_‐based film flexible thermoelectric device with vertical Y‐shaped configuration.^[^ ^56^ ^]^ First, n‐type Bi_2_Te_3_ and p‐type Sb_2_Te_3_ legs with the thickness around 100 μm were deposited on a precleaned Cr–Au seed layer using an electrochemical deposition method. Then, a Ti–TiN–Au–Cu multilayer was screen sputtered as interfacial contacts. Subsequently, Cu columns were electroplated on the Ti–TiN–Au–Cu multilayer as metallic interconnectors to conduct the heat from the skin vertically to the thermoelectric materials and then laterally through the thermoelectric materials. Finally, PDMS was used to fill the gaps among the p/n thermoelectric legs inside the device. An open‐circuit voltage of 56 mV and output power density of 30 mW m^−2^ were achieved when the device was attached to the human body (310 K).

**Figure 4:**
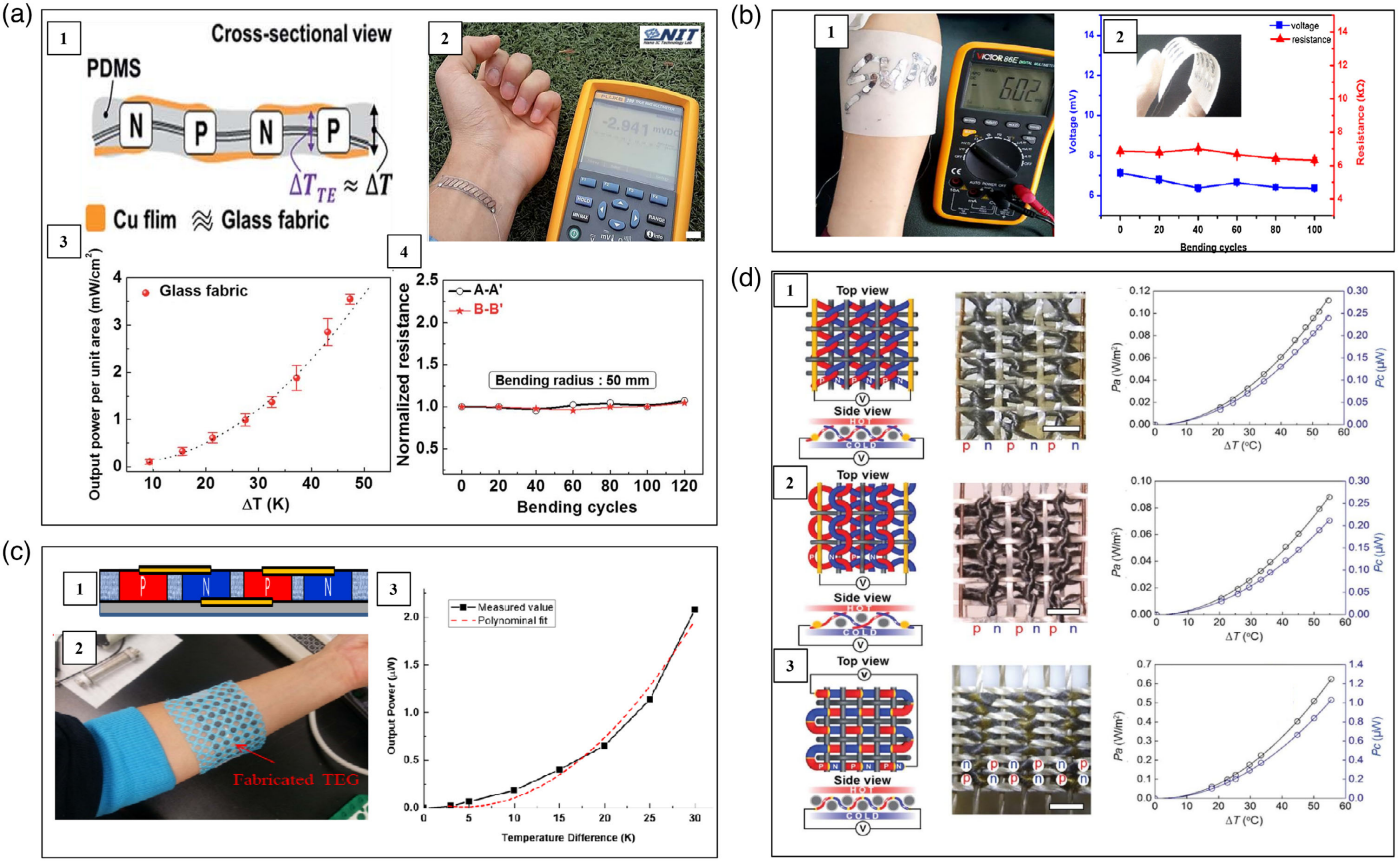
Schematic, optical image, and performance of several flexible thermoelectric devices consisting of Bi_2_Te_3_‐based alloys deposited on fabrics. a) An 8‐couple device on glass fabric. Reproduced with permission.^[^ ^7^ ^]^ Copyright 2014, Royal Society of Chemistry. b) A 12‐couple device on silk. Reproduced with permission.^[^ ^57^ ^]^ Copyright 2016, Elsevier. c) A 20‐couple device on silk. Reproduced with permission.^[^ ^58^ ^]^ Copyright 2013, IEEE. d) Thermoelectric textiles with different structures. Reproduced with permission.^[^ ^59^ ^]^ Copyright 2016, Wiley‐VCH.

Kim et al. prepared a fabric‐based flexible thermoelectric device adopting the vertical π‐shaped configuration by successively screen printing p‐type Sb_2_Te_3_ and n‐type Bi_2_Te_3_ pastes on the glass fabric.^[^ ^7^ ^]^ A 200 nm‐thick Ni interlayer was deposited on both sides of the thermoelectric legs as interfacial contacts between thermoelectric materials and the screen printed Cu electrodes. **Figure** **4**a shows the sketch map and the optical image of this device, which has a self‐sustaining structure without top or bottom substrates. Under Δ*T* = 50 K, this device demonstrated an open‐circuit voltage of 90 mV, a power density of 38 W m^−2^, and a power output per unit weight of 28 mW g^−1^. When attaching this device on human skin with an environmental temperature of 285 K, an open‐circuit voltage of 2.9 mV and an output power of 3 μW were achieved.

Lu et al. prepared p‐type Sb_2_Te_3_ and n‐type Bi_2_Te_3_ inks by hydrothermal method and then repeatedly deposited the corresponding inks on both sides of silk fabric (Figure 4b).^[^ ^57^ ^]^ The 12 p/n thermoelectric couples were electrically connected by Ag foils. Under Δ*T* = 35 K, the highest power output and open‐circuit voltage were ≈15 nW and ≈10 mV, respectively. Kim et al. fabricated one fabric‐based flexible thermoelectric device by using the dispenser printing technique to fill the mixture of ceramic binder and Bi_2_Te_3_ powders (p‐type and n‐type) into the windows of the polyester‐based fabric.^[^ ^58^ ^]^ The device includes 20 p/n couples. Ag‐contained conductive fabric fiber was used as electrodes between n‐type and p‐type legs. Under Δ*T* = 30 K, a maximum power output of 2.08 μW was achieved. As shown in Figure 4c, when the flexible thermoelectric device was attached to the human body, a power output of 178 nW was obtained in ambient temperature of 5 K.

Using Bi_2_Te_3_‐ and Sb_2_Te_3_‐PAN yarns, Lee et al. designed three kinds of flexible thermoelectric devices adopting vertical π‐shaped configuration with different structures shown in Figure 4d, termed zigzag‐stitch, garter‐stitch, and plain‐weave.^[^ ^59^ ^]^ Gold‐coated yarns were adopted as electrodes to connect n‐ and p‐type‐coated segments. Under Δ*T* = 55 K, the plain‐weave textile provided a higher power output (0.62 W m^−2^) than the textiles made by knitting individual n‐ and p‐type thermoelectric yarns with zigzag‐stitch (0.11 W m^−2^) and garter‐stitch structures (0.09 W m^−2^). The performance difference mainly came from the higher internal resistance in the zigzag‐stitch and garter‐stitch structures than that in the plain‐weave textile.

**Figure 5:**
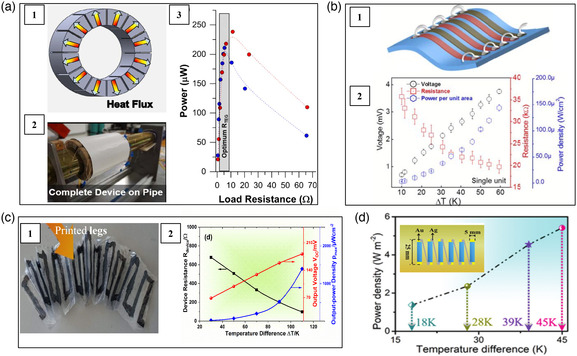
Schematic, optical image, and performance of several flexible thermoelectric devices consisting of a) printed Ni and Ag. Reproduced with permission.^[^ ^60^ ^]^ Copyright 2017, Elsevier. b) 1T‐TMDCs nanosheets. Reproduced with permission.^[^ ^61^ ^]^ Copyright 2016, Royal Society of Chemistry. c) Ag_2_Se. Reproduced with permission.^[^ ^63^ ^]^ Copyright 2020, American Chemical Society. d) Ag_2_Se/Ag/CuAgSe composites. Reproduced with permission.^[^ ^64^ ^]^ Copyright 2020, Royal Society of Chemistry.

Beyond the Bi_2_Te_3_‐based alloys, many other inorganic materials have been also used to fabricate flexible thermoelectric devices. Lezzi et al. designed a flexible thermoelectric device consisting of printed Ni and Ag as n‐type and p‐type legs, respectively.^[^ ^60^ ^]^ This device, including 420 p/n couples, was attached on a heat pipe. The sketch map and optical image of this device are shown in **Figure** **5**a. Under Δ*T* = 127 K, this device generated a power output of 308 μW. By introducing the boost converter to charge a pair of storage capacitors, this device can turn on a Bluetooth low energy enabled microcontroller used in a temperature sensing circuit and realize wireless communication capability. As shown in Figure 5b, Oh et al. fabricated a flexible device based on the chemical exfoliated 1T‐TMDCs nanosheets (n‐type WS_2_ and p‐type NbSe_2_).^[^ ^61^ ^]^ PDMS was used as the substrate and binding agent and Ag was used to realize electrical connection between the n‐type WS_2_ and p‐type NbSe_2_. The device produced a power output of 38 nW under Δ*T* = 60 K. Moreover, this device demonstrated good stability. Its performance was scarcely deteriorated even after 1000 bending cycles and 100 stretching cycles. Wang et al. fabricated a flexible thermoelectric device consisting of 4‐couple C_60_/TiS_2_ hybrid films as n‐type legs and (SWNTs)/PEDOT:PSS hybrid films as p‐type legs.^[^ ^62^ ^]^ The device adopting the lateral π‐shaped configuration generated a maximum power output of 335 nW and the normalized maximum power density of 1.68 W m^−2^ under Δ*T* = 20 K.

Ag_2_Se possesses good thermoelectric performance as n‐type commercial Bi_2_Te_3_‐based alloys near room temperature. Thus, many attempts have been made to fabricate Ag_2_Se‐based flexible thermoelectric device. Mallick et al. prepared Ag–Se‐based n‐type inks and commercial PEDOT:PSS p‐type inks and then used them to prepare a Ag_2_Se/PEDOT:PSS flexible device by the screen printing method.^[^ ^63^ ^]^ As shown in Figure 5c, polyethylene naphthalate (PEN) was used as substrate and Ag was used as the electrodes. The device adopting the lateral π‐shaped configuration generated an open‐circuit voltage of 181.4 mV and a power density of 3.21 mW m^−2^ under Δ*T* = 110 K. Lu et al. fabricated a Ag_2_Se‐based unipolar flexible thermoelectric device by using the n‐type flexible Ag_2_Se/Ag/CuAgSe composite film supported by nylon membrane (Figure 5d).^[^ ^64^ ^]^ Ag paste was used to connect the top and bottom sides of n‐type thermoelectric legs electrically in series. This device generated an open‐circuit voltage of 12.2 mV and a power output of 488 nW (power density ≈5.42 W m^−2^) under Δ*T* = 45 K.

The inorganic thermoelectric materials can be also fabricated into freestanding films without the supporting of substrate. For example, Paul et al. fabricated freestanding Ca_3_Co_4_O_9_ films with the thickness of 250 nm by using the reactive RF magnetron cosputtering and dry transfer technique.^[^ ^65^ ^]^ Although Ca_3_Co_4_O_9_ is intrinsically brittle, the gap network among the oriented nanolaminated Ca_3_Co_4_O_9_ grains can allow the film to sustain some degree of bending stress and gain some flexibility. The electrical transport properties of their freestanding Ca_3_Co_4_O_9_ films are comparable with those for bulk Ca_3_Co_4_O_9_. Moreover, the films show stable electrical transport properties after 100 times cycling bending test. These features endow the freestanding Ca_3_Co_4_O_9_ films great potential to be used in the flexible thermoelectric device.

#### 5.2.2 Deformable Interconnector‐Based

Via connecting the rigid bulks by the deformable interconnectors, such as flexible printed circuit board (FPCB), liquid metal electrodes, and polymer shafts, brittle inorganic thermoelectric materials can also realize some degree of flexibility in the device level. The advantage of this approach is that the inorganic thermoelectric materials can be directly cut from the large dense bulks fabricated by spark plasma sintering or hot pressing. In this case, the thermoelectric materials used in the flexible devices can well inherit the high‐density and excellent thermoelectric performance from the bulks. However, the main drawback of this approach lies on that the device's flexibility is dominated by the bonding at the interfaces between the interconnectors and thermoelectric materials. In the bending process, cracks are easily formed near the interfaces, which will result in low power output or even complete device failure. Thus, the application of deformable interconnector‐based flexible thermoelectric devices is limited in the conditions that require relatively small deformation.

**Figure 6:**
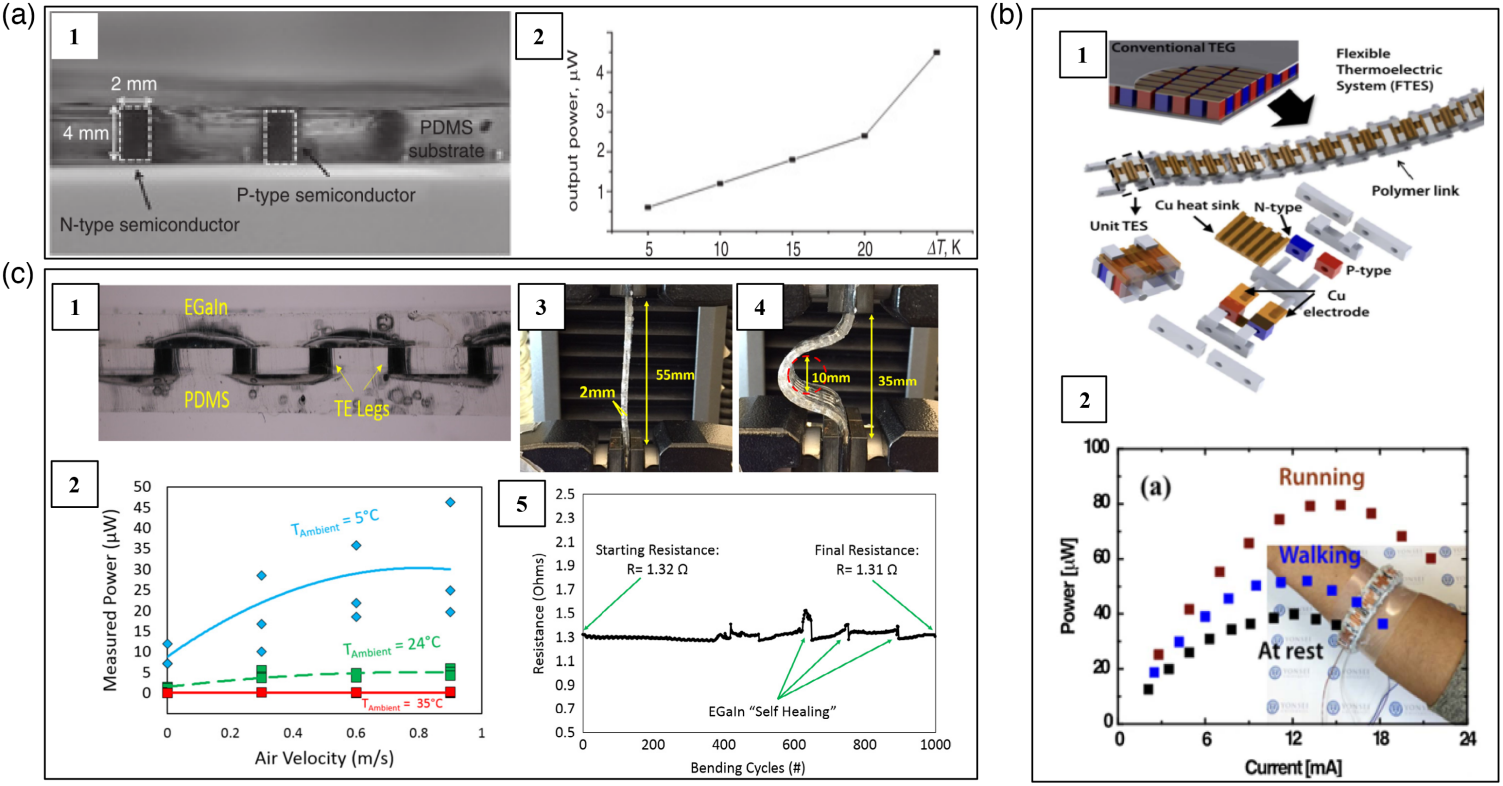
Schematic, optical image, and performance of several deformable interconnector‐based flexible thermoelectric devices consisting of Bi_2_Te_3_‐based alloys. a) A device connected by a FPCB. Reproduced with permission.^[^ ^66^ ^]^ Copyright 2012, Institution of Electrical Engineers. b) A 10–couple device connected by polymer shafts. Reproduced with permission.^[^ ^8^ ^]^ Copyright 2017, Elsevier. c) A 32‐couple device connected by EGaIn electrodes. Reproduced with permission.^[^ ^69^ ^]^ Copyright 2017, Elsevier.

Jo et al. fabricated an 8‐couple Bi_2_Te_3_‐based thermoelectric device encapsulated inside PDMS by using FPCBs as deformable interconnectors.^[^ ^66^ ^]^ P‐ and n‐type Bi_2_Te_3_ bulk legs with the height of 4 mm were inserted into the PDMS using the dispenser printing method. The Cu patterns on the FPCBs served as the electrodes between the thermoelectric legs. Due to the good deformable ability of PDMS and FPCBs, the device could achieve some degree of flexibility. An output power of 2.1 μW was achieved when it was attached to human skin at ambient temperature of 286 K (**Figure** **6**a). Kim et al. fabricated a self‐powered wearable electrocardiography using a Bi_2_Te_3_‐based flexible thermoelectric device adopting vertical π‐shaped configuration.^[^ ^67^ ^]^ The thermoelectric legs have an area of 4.0 mm^2^ and *L* of 2.5 mm. Cu foils were used as electrodes and deformable interconnectors. All these components were encapsulated in the PDMS. This device produced a power density of 386 mW m^−2^ when it is attached to the human wrist.

Eom et al. developed a spine‐shaped flexible thermoelectric device by using polymer shafts as deformable interconnectors to connect the p‐type Bi_0.5_Sb_1.5_Te_3_ and n‐type Bi_2_Te_2.7_Se_0.3_ thermoelectric legs.^[^ ^8^ ^]^ The dimension of each legs was 3 × 5 × 5 mm^3^. They were connected by 50 μm‐thick copper foil electrically in series. The assembled device consisted of 40 p/n couples. As shown in Figure 6b, this biomimetic structure endowed the device good flexibility to attach onto curved heat sources. They tested the power output of the device in different conditions. When a person was at rest, walking, and jogging, the power outputs were ≈40, 50, and 80 μW, respectively.

In the bending cycle test, the interface between the thermoelectric materials and the traditional metallic interconnectors is easily cracked, which can lead to the significant power output deterioration or complete device failure. EGaIn, the eutectic alloy of gallium (Ga) and indium (In), can withstand large bending without broken. Particularly, EGaIn has the self‐healing ability. Thus, EGaIn is a better deformable interconnector than traditional FPCBs or Cu foils. It can greatly lessen the stress at the interface. Zadan et al. used the liquid metal EGaIn as deformable interconnectors to fabricate the Bi_2_Te_3_‐based flexible thermoelectric device.^[^ ^68^ ^]^ Under Δ*T* = 60 K, the device generated a voltage of 278.6 mV and a power density of 866 mW m^−2^. Suarez et al. fabricated a similar module using the liquid metal EGaIn as deformable interconnectors,^[^ ^69^ ^]^ which not only provided extremely low contact resistance (4 μΩ cm^2^) but also good stretch ability and self‐healing ability. When the device was worn on human wrist, this device generated a maximum voltage of 8.22 mV and a power output of 46.28 μW at the ambient temperature of 278 K and air velocities of 0.9 m s^−1^. As shown in Figure 6c, after 1000 times bending cycles, the internal resistance of the device (1.31 Ω) was almost the same with the starting total resistance (1.32 Ω). Sargolzaeiaval et al. fabricated a Bi_2_Te_3_‐based flexible thermoelectric device in which the Bi_2_Te_3_ bulks were embedded inside a stretchable and high thermal conductivity elastomer consisting of PDMS with graphene nanoplatelets/EGaIn inclusions.^[^ ^70^ ^]^ EGaIn liquid metal was used to connect the bulk Bi_2_Te_3_ thermoelectric legs electrically in series. This device achieved a power density in excess of 0.3 W m^−2^ when it was worn on wrist at an air velocity of 1.2 m s^−1^.

### 5.3 Organic/Inorganic Composites/Hybrids‐Based

Due to the low‐carrier‐mobility hopping charge carriers, the organic materials generally possess high electrical resistivity. Compositing high‐carrier‐mobility low‐dimensional inorganic materials (e.g., metallic nanowires, CNTs, graphene, Ta_4_SiTe_4_ whiskers, or Bi_2_Te_3_ nanosheets) and organic materials at the micrometer and/or millimeter scale can effectively lower the electrical resistivity by establishing charge carrier conduction paths across the composites, yielding greatly enhanced power factors while still maintaining the good flexibility. Furthermore, compositing the inorganic materials and organic materials at the nanometer or even molecular scale can generate flexible hybrid materials which might display new properties beyond those observed for the original matrix materials. These flexible organic/inorganic composites/hybrids provide more options to fabricate flexible thermoelectric devices.

**Figure 7:**
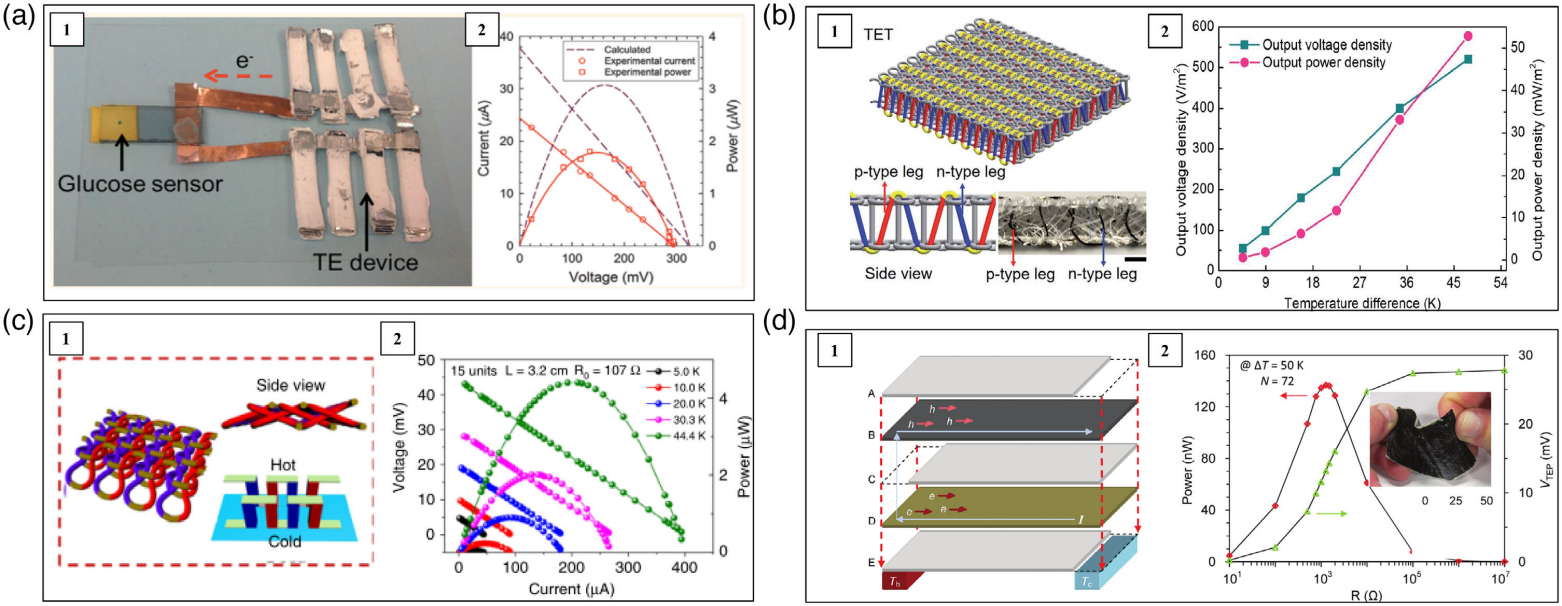
Schematic, optical image, and performance of several CNTs‐based flexible thermoelectric devices. a) A 72‐couple device adopting lateral π‐shaped configuration. Reproduced with permission.^[^ ^72^ ^]^ Copyright 2014, American Chemical Society. b) A 966‐couple knitted thermoelectric textile. Reproduced with permission.^[^ ^76^ ^]^ Copyright 2020, Royal Society of Chemistry. c) A 15‐couple thermoelectric textile. Reproduced under the terms of the CCȐBY 4.0 license.^[^ ^77^ ^]^ Copyright 2020, The Authors, published by Springer Nature. d) A 36‐couple multilayered device. Reproduced with permission.^[^ ^78^ ^]^ Copyright 2012, American Chemical Society.

Wang et al. fabricated an unipolar flexible thermoelectric device by using single‐walled carbon nanotubes (SWNTs)/PANI composites as p‐type legs.^[^ ^71^ ^]^ Due to the formation of a highly ordered structure, the SWNTs/PANI composites demonstrated low electrical resistivity in the order of 10^−6^ Ωm and high power factor of 217 μW m^−1^ K^−2^ at 300 K. Silver paste and gold wires were used as electrodes. This unipolar device adopting the lateral π‐shape configuration generated an open voltage of 8.2 mV and a power output of 3.76 mW under Δ*T* = 60 K. Kim et al. prepared a flexible thermoelectric device based on CNTs/organic composites adopting lateral π‐shaped configuration (**Figure** **7**a).^[^ ^72^ ^]^ They first treated CNTs by using polyethylenimine, diethylenetriamine, and NaBH_4_ to realize n‐type conduction and sodium dodecyl‐benzenesulfonate (SDBS) to obtain p‐type conduction, respectively. Then, polytetrafluoroethylene (PTFE) membrane was used to prepare large p‐ and n‐type CNT‐based films. The obtained CNTs films were cut into thermoelectric legs with the dimension of 25 × 4 mm^2^. Al foil was used as electrodes to connect the n‐ and p‐type thermoelectric legs. The fabricated device including 72 p/n couple showed an open‐circuit voltage of 465 mV under Δ*T* = 49 K. This device could operate a glucose sensor at a temperature difference of 32 K. Cho et al. fabricated a unipolar thermoelectric device adopting lateral π‐shaped configuration. PANi/graphene‐PEDOT:PSS/PANi/DWNT‐PEDOT:PSS composites in the form of thin film were used as p‐type legs and they were deposited on a PET substrate.^[^ ^73^ ^]^ Ag wires were used as n‐type legs. At an ambient temperature of 298.6 K and Δ*T* = 9.7 K, this device generated an open‐circuit voltage of 5.10 mV and a power output of 8.5 nW. Hong et al. prepared a p‐type CNT/poly(3‐hexylthiophene) (CNT/P3HT) nanocomposite films by spray‐printing method.^[^ ^74^ ^]^ This device generated a maximum power output of 32.7 nW under Δ*T* = 10 K.

Zhou et al. cut the p‐type CNT films into strips and placed them onto the PET substrate.^[^ ^75^ ^]^ Then, the solution of polyethyleneimine and ethanol was drop‐casted into specific areas on the CNT strips to form n‐type legs. Ag paste was used as the electrodes. The corresponding 3‐couple flexible thermoelectric device produced a voltage of 10 mV and a power of 2.5 μW under Δ*T* = 27.5 K. Zheng et al. fabricated a spacer fabric shaped thermoelectric textile using silver paste‐connected CNTs yarn shown in Figure 7b.^[^ ^76^ ^]^ First, CNTs yarn was wrapped onto a thin polyethylene terephthalate (PET) plate and rolled up to a packed cylindrical structure. Then, Ag paste was applied to draw two parallel lines directly on the CNTs yarn. Subsequently, it was alternately immersed into aqueous PEDOT:PSS solution and PEI/ethanol solution to get p‐type legs and n‐type legs, respectively. Finally, the yarn was twisted with a polyester filament to enhance its mechanical stability. This device generated a power density of 515 W m^−2^ under Δ*T* = 47.5 K. Using electrospray technology, Sun et al. also fabricated CNTs yarn‐based thermoelectric textiles (Figure 7c).^[^ ^77^ ^]^ The n‐type legs were obtained by doping oleamine, while the p‐type legs were obtained by doping PEDOT:PSS. The CNTs without doping were used as electrodes to connect the n‐ and p‐type legs. The CNTs yarns were wrapped by acrylic fibers and a power output of 70 mW m^−2^ was generated under Δ*T* = 44 K. Hewitt et al. developed a CNTs‐based flexible thermoelectric device with a sandwich structure shown in Figure 7d.^[^ ^78^ ^]^ N‐type CNTs/PVDF conduction layer, PVDF insulation layer, and p‐type CNTs/PVDF conduction layer were alternately placed into a stack. The stack was pressed and heated to 450 K to melt the PVDF and bond the stacking layers. As the n‐ and p‐type CNTs were directly contacted with each other at the edges of the stacking layers, this structure did not require extra electrodes. A power output of 137 nW was obtained under Δ*T* = 50 K.

Beyond CNTs, Chen et al. fabricated Co nanowires/PVDF nanocomposites wherein the Co nanowires were selectively oriented by applying a magnetic field.^[^ ^79^ ^]^ The composites demonstrated a high power factor of 523 μW m^−1^ K^−2^ at 320 K. By using these nanocomposites as n‐type legs and commercial PEDOT:PSS thin films as p‐type legs, a 10‐couple flexible thermoelectric devices adopting lateral π‐type configuration was fabricated onto a flexible polyimide substrate. Ag paste was used as electrodes. The device generated a maximum power output of 5.2 μW under Δ*T* = 50 K, corresponding to the power density of 1 μW mg^−1^ and 0.26 μW cm^−2^.

Being limited by the metallic transport properties of CNTs or metal nanowires, the organic/inorganic composites/hybrids including CNTs or metal nanowires usually possess low Seebeck coefficient. Addressing this issue, some low‐dimensional inorganic semiconductors with intrinsically large Seebeck coefficient were also used to make flexible organic/inorganic composites/hybrids. See et al. synthesized Te nanowires/PEDOT:PSS composite films by drop‐casting method and achieved a *zT* around 0.1 at room temperature.^[^ ^80^ ^]^ Du et al. prepared Bi_2_Te_3_ nanosheet/PEDOT:PSS composites by adopting spin coating and drop‐casting techniques. The film shows a *PF* value around 32.26 μW m^−1^ K^−2^ at room temperature.^[^ ^81^ ^]^ Zhang et al. incorporated nanosized Bi_2_Te_3_ particles into PEDOT:PSS. By using the HCl solution to remove the surface oxidation layer of Bi_2_Te_3_ particles, a *PF* value of 131 μW m^−1^ K^−2^ was achieved in the p‐type Bi_2_Te_3_/PEDOT:PSS composite at ambient temperature.^[^ ^82^ ^]^ Xu et al. composited semiconducting Ta_4_SiTe_4_ whiskers that has intrinsically large *S* and high *PF* with PVDF.^[^ ^83^ ^]^ The Ta_4_SiTe_4_/PVDF composites exhibit good flexibility and excellent electrical performance superior to most previously reported flexible organic/inorganic thermoelectric composites. The flexible thermoelectric device consisting of Ta_4_SiTe_4_/PVDF composites generated a maximum power output of 1.68 mW under Δ*T* = 35.5 K.

In addition to the normal composites/hybrids, some organic small molecules can be intercalated into inorganic layered TiS_2_ compound forming hybrid superlattice structures. Typical examples are TiS_2_/[(hexylammonium)_*x*_(H_2_O)_*y*_(DMSO)_*z*_], TiS_2_[tetrabutylammonium]_*x*_[hexylammonium]_*y*_, and TiS_2_/hexylamine.^[^ 84, 85 ^]^ In these hybrid superlattice structures, inorganic TiS_2_ atomic layers provide electrical transport paths for high *PF*, while the intercalated organic molecular layers strengthen the phonon scattering yielding low *κ*. A high *zT* value of 0.28 was achieved for TiS_2_/[(hexylammonium)_*x*_(H_2_O)_*y*_(DMSO)_*z*_] at 373 K.^[^ ^84^ ^]^ A single‐leg device based on TiS_2_/hexylamine superlattice generated a maximum power output of 24 nW and a power density of 32 μW cm^−2^ under Δ*T* = 20 K.^[^ ^86^ ^]^

### 5.4 Plastic Deformable Inorganic Semiconductors‐Based

Inorganic semiconductors are generally brittle at room temperature. They are prone to break under large deformation and tension. For a long time, it is considered that the inorganic thermoelectric materials with intrinsic plastic deformability do not exist at room temperature and the inorganic thermoelectric materials can only gain some elastic deformation in the special forms such as low‐dimension films. Till most recently, this thought was subverted by the discovered abnormal plastic deformability in bulk Ag_2_S polycrystal,^[^ ^11^ ^]^ InSe single crystal,^[^ ^42^ ^]^ and ZnS single crystal in darkness.^[^ ^41^ ^]^ On one hand, comparing with the brittle inorganic semiconductors, these plastic deformable semiconductors are easy to be machined into flakes/films to fabricate the flexible devices.^[^ ^87^ ^]^ On the other hand, although the flexible devices fabricated by these plastic deformable inorganic semiconductors are also used in the elastic (reversible) regime, the material's intrinsically plastic deformability can endow them larger magnitude of flexibility than those fabricated by the brittle semiconductors. Thus, these plastic deformable inorganic semiconductors provide a new avenue to fabricate flexible thermoelectric materials and devices.

**Figure 8:**
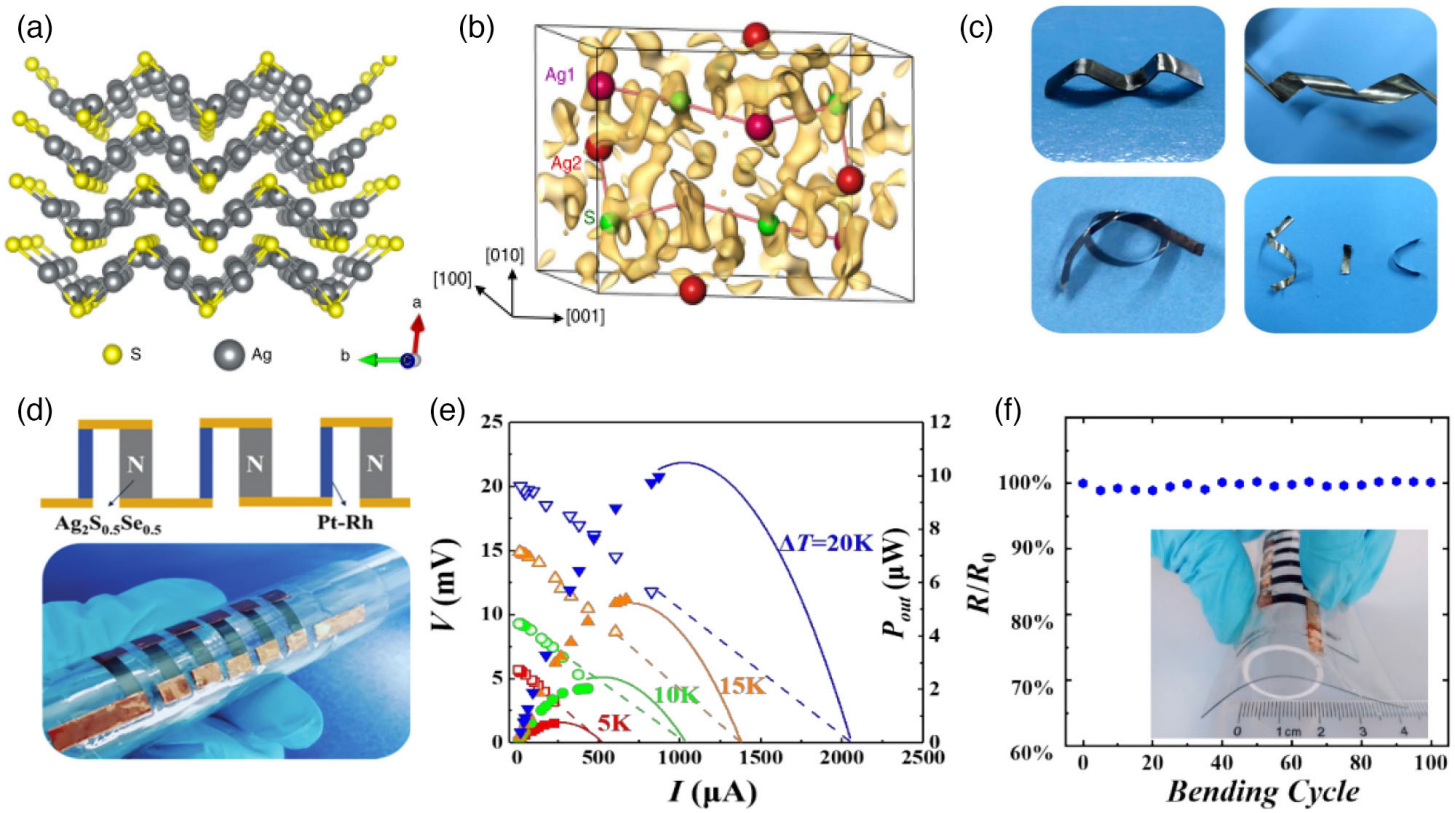
a) Perspective view of the Ag_2_S crystal structure along the [001] direction. b) Enlarged zigzag fragment of two adjacent atomic layers. a,b) Reproduced with permission.^[^ ^11^ ^]^ Copyright 2018, Springer Nature. c) Ag_2_S‐based compounds twisted into different shapes. d) Schematic and optical image of a 6‐couple flexible Ag_2_S_0.5_Se_0.5_/Pt‐Rh TE device. e) Output voltage *V* and power output *P*_out_ as a function of current (*I*) for this device under different operating temperature difference. f) Stability test showing that the relative electrical resistance variation *R/R*_0_ of this device is scarcely changed after bending various number of times. The inset shows the optical image for the bended device. c–f) Reproduced with permission.^[^ ^12^ ^]^ Copyright 2019, Royal Society of Chemistry.

Recently, Ag_2_S‐based flexible thermoelectric materials and devices have been studied and developed. Ag_2_S is a typical semiconductor with a bandgap of 1.03 ± 0.1 eV at room temperature. The Ag_2_S ingot exhibits a maximal deformation about 50% engineering strain in the compression tests and an engineering strain above 20% without cracking in the three‐point bending tests.^[^ ^11^ ^]^ Thus, Ag_2_S is different with most inorganic semiconductors reported before. It demonstrates abnormal metallic‐like plastic deformability. **Figure** **8**a shows the crystal structure of α‐Ag_2_S along the [001] direction. It has a stacked layered structure along the *a*‐axis. As shown in Figure 8b, the S atoms in the upper layer are always bonded to part of the Ag atoms in the lower layer during the slipping process. The multicentered, diffuse and relatively weak bonding in Ag_2_S gives rise to the small slipping energy and large cleavage energy, i.e., facilitating the slipping without cleavage, which are responsible for the abnormal metallic plastic deformability of Ag_2_S.

The carrier mobility for Ag_2_S is around 100 cm^2^ V^−1^ s^−1^ at room temperature, but its low carrier concentration, in the order of 10^14^ cm^−3^ at room temperature, limits it to achieve high electrical conductivity and good thermoelectric performance. Via alloying Se and/or Te in Ag_2_S to reduce the defect formation energy of Ag interstitial atom, the carrier concentration can be significantly enhanced by several orders of magnitude, yielding greatly enhanced thermoelectric performance. A *zT* value of 0.26 and 0.39 was obtained for Ag_2_S_0.5_Se_0.5_^[^ ^12^ ^]^ and Ag_20_S_7_Te_3_^[^ ^88^ ^]^ at 300 K, respectively. Further alloying Te in Ag_2_S_0.5_Se_0.5_ can result in a higher *zT* value of 0.44 at 300 K. These *zT* values are comparable with or higher than those of organic thermoelectric materials. More importantly, these Se and/or Te alloyed Ag_2_S‐based compounds still maintain good plastic deformability and flexibility as Ag_2_S.^[^ ^89^ ^]^ As shown in Figure 8c, they can be twisted into different kinds of shapes to adapt the curved heat sources.

Due to the intrinsic plastic deformability and flexibility, Ag_2_S‐based compounds can be directly machined from bulks into flexible thin foils without the support of flexible substrates or deformable interconnectors. Thus, the fabrication of flexible thermoelectric devices consisting of Ag_2_S‐based compounds is much simpler than those consisting of Bi_2_Te_3_‐based alloys. As shown in Figure 8d, by using the thin foils directly cut from an Ag_2_S_0.5_Se_0.5_ ingot, a 6‐couple Ag_2_S‐based all‐inorganic flexible thermoelectric device adopting lateral π‐shaped configuration was fabricated. Pt–Rh wires were used to connect the top and bottom ends of n‐type thermoelectric legs electrically in series. This device generated a power output of 10 μW under Δ*T* = 20 K (shown in Figure 8e). Moreover, this device showed good mechanical stability during cycling bent test. As shown in Figure 8f, upon 100 bending cycles at a bending radius of 10 mm, the electrical resistance of the device was scarcely changed. This study provided a testimony to the feasibility of all‐inorganic flexible thermoelectric generators for wearables.

## 6 Summary and Outlooks

In the past decade, flexible thermoelectrics has achieved notable progress. High *zT*s have been reported in various new flexible organic thermoelectric materials and many new fabrication methods have been developed to endow the high‐performance inorganic thermoelectric materials good flexibility in device level. Moreover, the inorganic thermoelectric materials with intrinsic plastic deformability have been also discovered. Lots of flexible thermoelectric devices with different configurations have been fabricated, showing the ability to generate nW‐level or even μW‐level electricity by using the small temperature difference (**Table** **1**). Some of the devices have already demonstrated the ability to power the small electronics (e.g., glucose sensor, pulse oximeter, and electroencephalogram^[^ ^90^ ^]^) in the wearables. Moreover, the applications of flexible thermoelectric materials and devices are not limited in the power supply for wearables. They can be also integrated into the electronic skin to detect the environmental thermal stimuli, or into the virtual reality (VR) wearable devices to create cold or hot feelings and bring better immersive experience to the wearers.

**Table 1:** Summary of the performance of typical flexible thermoelectric devices listed in this review

Year	Type	Heat resource	Materials	Δ*T* [K]	Power density	Leg height *L*	*P•L/A/*Δ*T* ^*2*^[μW m^−1 ^K^−2^]	DOI
2011	Lateral	–	PEDOT–Tos + TTF‐TCNQ	30	0.27 μW cm^−2^	30 μm	9 × 10^−5^	10.1038/NMAT3012
2012	Vertical	–	Poly[Na_*x*_(Ni‐ett)] Poly[Cu_*x*_(Cu‐ett)]	80	2.8 μW cm^−2^	0.9 mm	0.004	10.1002/adma.201104305
2012	Lateral	–	BST	20	130 μW cm^−2^	5 mm	16.25	10.1021/am301759a
2012	Vertical	–	PEDOT‐Tos/TTF‐TCNQ	30	1.2 μW cm^−2^	0.9 mm	0.012	10.1039/c2ee22777k
2013	Lateral	–	BST	20	0.139 mW cm^−2^	2 cm	69.5	10.1002/smll.201301025
2014	Lateral	–	BST	70	130 μW cm^−2^	5 mm	1.33	10.1063/1.4861057
2014	Vertical	–	BST	50	3.8 mW cm^−2^	500 μm	7.6	10.1039/c4ee00242c
2016	Vertical	–	BST	25	4.78 mW cm^−2^	650 μm	49.712	10.1021/acsnano.6b05004
2016	Textile	–	BST	55	8.56 W m^−2^	2 mm	5.66	10.1002/adma.201600709
2016	Lateral	Wrist	WS_2_ (n‐type) + NbSe_2_ (p‐type) NS	–	0.73 × 10^−3^ μW cm^−2^	–	–	10.1039/c5ee03813h
2017	A single film leg	–	CuI	50	2.4 mW cm^−2^	–	–	10.1038/ncomms16076
2017	Lateral	Wrist	CNT	–	0.125 × 10^−3^ μW cm^−2^	–	–	10.1039/c7ta00304h
2018	Y‐type	Human body	BST	–	3 μW cm^−2^	–	–	10.1016/j.apenergy.2017.05.005
2018	Vertical	Wrist	BST	–	38 μW cm^−2^	2.5 mm	–	10.1021/acsenergylett.7b01237
2018	Lateral	–	PEDOT:PSS + C60/TiS_2_	20	1.68 W m^−2^	10 mm	42	10.1039/c7ee03617e
2018	Lateral	–	Co nanowires/PVDF + PEDOT:PSS	50	0.26 μW cm^−2^		–	10.1002/aelm.201800200
2019	Lateral	–	Ag_2_Se	30	2.3 W m^−2^	20 mm	51.1	10.1038/s41467‐019‐08835‐5
2019	Lateral	–	Ag_2_(S, Se)	20	5.33 W m^−2^	15 mm	200	10.1039/c9ee01777a
2019	Lateral	–	Ag_2_Se/Ag/CuAgSe	45	5.42 W m^−2^	20 mm	53.53	10.1039/c9ee01609k
2020	Lateral	–	Ag_2_Se	110	321 μW cm^−2^	–	–	10.1021/acsami.0c01676
2020	Lateral	–	BST	60	1.4 mW cm^−2^	15 mm	58.33	10.1021/acsami.9b21771
2020	Vertical	–	BST	60	86.6 μW cm^−2^	3 mm	0.723	10.1021/acsami.9b19837
2020	Vertical	Wrist	BST	–	30 μW cm^−2^	3 mm	–	10.1016/j.apenergy.2019.114370
2020	Lateral	–	(PVDF)/Ta_4_SiTe_4_	35.5	13 W m^−2^	10 mm	103.15	10.1039/c9ee03776d
2020	Textile	–	CNT	47.5	51.5 mW m^−2^	–	–	10.1039/c9ta12494b
2020	Textile	–	CNT	44	70 mW m^−2^	32 mm	1.157	10.1038/s41467‐020‐14399‐6
2020	Vertical	–	BST	2.37	90.47 nW cm^−2^	245 μm	0.0395	10.1002/admt.202000486
2020	Vertical	–	PEDOT:PSS (p) + doped fullerene derivative(n)	25	30.5 nW cm^−2^	25 μm	0.0000122	10.1016/j.nanoen.2020.104983

**Figure 9:**
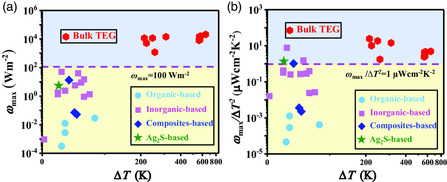
a) Maximum power density (*ω*_max_) and b) normalized maximum power density (*ω*_max_/Δ*T* ^2^) for typical flexible thermoelectric devices consisting of organic materials, traditional brittle inorganic materials, organic/inorganic composites, and plastic deformable Ag_2_S‐based compounds. The data for traditional rigid bulk devices are included for comparison. The data are taken from refs.^[^ 7, 12, 50, 52, 59, 62, 64, 68, 70, 76, 77, 83, 91, 92, 93, 94, 95, 96, 97, 98, 99, 100, 101, 102, 103, 104, 105, 106, 107, 108 ^]^

Despite the extensive research on flexible thermoelectrics in the past decade, it should be noted that currently they are still far away the large‐scale applications. One of the main reasons lies on the low power density generated by the flexible thermoelectric devices. As shown in **Figure** **9**a, the power density of reported flexible thermoelectric devices is generally lower than those of traditional bulk thermoelectric devices. Even if we use the normalized maximum power density (*ω*_max_/Δ*T* ^*2*^, with the unit of μW cm^−2^ K^−2^) to plot the data (Figure 9b), the difference is still very obvious. The poor power density of flexible thermoelectric devices is expected to be mainly originated from two aspects. On one hand, the flexible thermoelectric materials used in the flexible thermoelectric devices usually possess poorer *PF* and *zT* than those used in the traditional rigid thermoelectric devices. The thermoelectric materials used in the flexible thermoelectric devices are usually prepared by deposition or printing methods. Comparing with the bulks experienced long‐term annealing process and consolidation process, the deposited or printed materials might possess low density, poor crystallinity, or unoptimized carrier concentration, which would lead to poor thermoelectric performance and consequently low power density in device level. On the other hand, the power density of flexible thermoelectric devices is more sensitive to the electric/thermal contact resistance near the interfaces. For most traditional bulk thermoelectric devices with the thickness *L* in the cm level or at least mm level, the deterioration in the power density from the electric contact resistivity can be neglected when it is less than 10 μΩ cm^2^. However, to ensure the flexibility in the flexible thermoelectric devices based on traditional inorganic thermoelectric materials, the thermoelectric legs are usually less than 100 μm or even 10 μm. In this case, very small electric contact resistivity is enough to induce the great deterioration on the power density. This is believed to be responsible for the low power density of most flexible organic thermoelectric devices shown in Figure 9.

Based on the aforementioned understanding, improving the flexible thermoelectric materials’ performance and reducing the electric/thermal contact resistances at the interfaces are two most important ways to enhance the power density of flexible thermoelectric devices. Beyond these, theoretical structure design is also necessary for the flexible thermoelectric devices. Regarding the tiny temperature difference between the human body and environment, the curve surface of skin, and complex heat flux distribution, the structure of flexible thermoelectric devices (e.g., leg dimension, leg number, fill factor, filler materials among the legs, and substrate thickness) must be well designed to sufficiently use the heat generated by human body. In addition, because the flexible thermoelectric devices might experience frequently bending or stretching process during service, the mechanical performance, especially the rigidity near the interfaces area, should be also paid attention on. Currently, Ag paste is usually used to connect the thermoelectric materials and electrodes, but the mechanical performance is rarely investigated. Moreover, as the power out generated by the flexible thermoelectric devices is usually just nW level or μW level, the high‐efficient power management is indispensable.

Last but not the least, the cost is also one key factor that influences the large‐scale applications of flexible thermoelectrics. The thermoelectric materials should be cheap and the fabrication process should be easy to realize mass production with low cost. The organic thermoelectric materials and devices usually have poorer performance but lower cost than the inorganic thermoelectric materials and devices. Likewise, the inorganic thermoelectric films and flexible thermoelectric devices fabricated by the physical methods (e.g., molecular beam epitaxy, pulsed laser deposition, and magnetron sputtering) usually possess higher density and better quality but higher cost than those fabricated by the printing techniques (e.g., inkjet printing, dispenser printing, and screening printing). Thus, a trade‐off between performance and cost is required for flexible thermoelectrics in the real applications.

To sum up, the development of flexible thermoelectrics is more complicated than that of traditional thermoelectrics. This not only requires extensive research work conducted in laboratories, but also lots of additional technical and engineering studies that go beyond. Currently, more and more researchers from different fields are taking part in the development of flexible thermoelectric materials and devices. It is expected that flexible thermoelectrics would make meaningful contributions in the wearables in the near future.

## Conflict of Interest

The authors declare no conflict of interest.
